# Scaling functional status within the interRAI suite of assessment instruments

**DOI:** 10.1186/1471-2318-13-128

**Published:** 2013-11-21

**Authors:** John N Morris, Katherine Berg, Brant E Fries, Knight Steel, Elizabeth P Howard

**Affiliations:** 1 Institute for Aging Research, Hebrew SeniorLife, 1200 Centre Street, Boston, MA 02131, USA; 2 University of Toronto, 160-500 University Avenue, Toronto, ON M5G 1 V7, Canada; 3 School of Public Health, Geriatric Research, Education and Clinical Center, Ann Arbor VA Healthcare Center, University of Michigan, 300 NIB, 933 NW, Ann Arbor, MI 48109, USA; 4 Hackensack University Medical Center, 30 Prospect Avenue, Hackensack, NJ 07601, USA; 5 Bouvé College of Health Sciences, School of Nursing, Northeastern University, 360 Huntington Avenue, Boston, MA 02115, USA

## Abstract

**Background:**

As one ages, physical, cognitive, and clinical problems accumulate and the pattern of loss follows a distinct progression. The first areas requiring outside support are the Instrumental Activities of Daily Living and over time there is a need for support in performing the Activities of Daily Living. Two new functional hierarchies are presented, an IADL hierarchical capacity scale and a combination scale integrating both IADL and ADL hierarchies.

**Methods:**

A secondary analyses of data from a cross-national sample of community residing persons was conducted using 762,023 interRAI assessments. The development of the new IADL Hierarchy and a new IADL-ADL combined scale proceeded through a series of interrelated steps first examining individual IADL and ADL item scores among persons receiving home care and those living independently without services. A factor analysis demonstrated the overall continuity across the IADL-ADL continuum. Evidence of the validity of the scales was explored with associative analyses of factors such as a cross-country distributional analysis for persons in home care programs, a count of functional problems across the categories of the hierarchy, an assessment of the hours of informal and formal care received each week by persons in the different categories of the hierarchy, and finally, evaluation of the relationship between cognitive status and the hierarchical IADL-ADL assignments.

**Results:**

Using items from interRAI’s suite of assessment instruments, two new functional scales were developed, the interRAI IADL Hierarchy Scale and the interRAI IADL-ADL Functional Hierarchy Scale. The IADL Hierarchy Scale consisted of 5 items, meal preparation, housework, shopping, finances and medications. The interRAI IADL-ADL Functional Hierarchy Scale was created through an amalgamation of the ADL Hierarchy (developed previously) and IADL Hierarchy Scales. These scales cover the spectrum of IADL and ADL challenges faced by persons in the community.

**Conclusions:**

An integrated IADL and ADL functional assessment tool is valuable. The loss in these areas follows a general hierarchical pattern and with the interRAI IADL-ADL Functional Hierarchy Scale, this progression can be reliably and validly assessed. Used across settings within the health continuum, it allows for monitoring of individuals from relative independence through episodes of care.

## Background

As one ages, there comes a point when the support of others is not only a social nicety but also a functional necessity. Physical, cognitive, and clinical problems accumulate over time and tasks that once could be done without the help of others become challenging or impossible to perform. Since the earliest work of Katz, functional loss has shown to follow a distinct progression [1]. Typically, the first areas requiring outside support are the Instrumental Activities of Daily Living (IADLs), for example, cooking, cleaning, managing medications, and shopping. These are basic functions no matter where one lives, although the subtasks involved in each area can vary depending on the setting in which one lives (e.g., turning on a water tap or stove burner in Toronto, as against gathering wood and carrying water to the home in an urban township outside of Cape Town). The age of onset and pace of loss also will vary from one person to the next, but as time progresses, there typically becomes a time when one needs the support of others in performing the more Activities of Daily Living (ADLs), i.e., dressing, personal hygiene, walking, transferring, toileting, and eating. A variety of functional items and summary scales exist for providing the context to describe this movement from independence to full dependency [1-6]. One such set of functional items created by the international interRAI non-profit consortium is included in its multi-setting suite of assessment instruments [7,8]. This set of instruments is in wide use across the world, mandated in many instances by local, state, and national governments.

Such scales and instruments describe persons living in diverse community settings, with different underlying disease and family support situations. In an earlier work, interRAI described a set of ADL summary scales [4], scales that are in wide use across the world. We envision the new development described here will enjoy similar use and dissemination. These measures will present some of the most effective summaries of the person’s status, providing useful feedback to the assessor, care planner, physician, and even the assessed person him/herself.

In this paper two new functional hierarchal scales are presented. The first is an IADL hierarchical capacity scale. This scale is based on items that reference how well the person could perform IADL tasks if called upon to do so. Capacity rather than actual performance is the key. Performance estimates are clouded by shared task assignments (e.g., two members of a couple perform different IADL tasks) and societal norms as to who should perform a task (e.g., housework or meal preparation).

The second of the new scales is a combination hierarchical scale integrating both the new IADL hierarchy and the established ADL hierarchy. This scale captures the full profile of loss in function from the earliest IADL in which help is required to the last ADL in which the person retains some ability to remain engaged in the task. A combination IADL-ADL hierarchical scale permits a single measure that tracks progressive loss across the full spectrum of functional tasks. It can be used across settings within the health continuum and allows for monitoring of individuals from relative independence through complex end-stage episodes of care.

A set of functional items created by interRAI is included in all of the assessment instruments in its multi-setting suite [4,7,8]. The functional items are assessed over a specific time period (3 days), each task has a set of specific sub-tasks noted (which could vary to adhere to local lifestyle profiles), and each item uses the same defined behavioral response options. Each of these items has been shown to have excellent inter-rater reliability when tested in countries across the world [9,10]. Furthermore, a substantial body of work has shown the relationship between these items and other functional and outcome measures. For example, the ADL items comprise the core measures for the widely used nursing home Resource Utilization Groups [RUGs] [11], while the IADL and ADL items are used in the home care version of the RUGs [12]. A decline in these items has been shown to be associated with cognitive decline [13]. Further, they are crucial components of screeners to forecast the level of care needs of elders in the community [14] and to create program-level quality indicators [15,16].

In creating functional scales, it has been recognized that there is a typical order of progressive functional loss [1]. For example, in the ADL domain, support with personal hygiene will be required before assistance with eating. Also, a progressive decline in the ability to perform the instrumental tasks precedes a decline in ADLs [17], although the pattern of loss among the specific IADLs is not as distinct as that which has been reported for ADLs.

The movement to summarize these items in reliable and consistent scales fills a variety of needs. At the clinical level they provide a foundation for assessing the person’s status and the types of support resources required. Perhaps even more importantly, they provide a continually updated baseline for assessing change in the months that follow the baseline assessment. Also they can be used at a programmatic level to assess intervention effectiveness and help with decisions about the allocation of resources within a program. The ADL Hierarchy Scale has been used extensively to describe and categorize persons when making comparisons across home care and long-term care settings internationally [13,18].

The interRAI functional assessment model is facilitated through the development and publication of assessment support manuals, providing explicit definitions and guidance on all of the items assessed [7]. Furthermore interRAI recommends an approach to training which is in wide use and, when followed, results in a consistent reliable assessment [9,10].

The IADL and ADL item sets are broad in scope, capturing a diverse array of functional tasks that are crucial to daily living. When able to be performed by the person, continued community residency can be expected; as deficits mount, transition to residency with others, supportive housing, and (where available) long-term care facilities must be contemplated. For example, the ability to live on one’s own almost always requires independence in IADL capacity for meal preparation (assembling the ingredients, cooking the food, and setting it out once prepared). It also requires the ability to carry out routine housework, including tasks such as sweeping the floor, washing dishes, making a bed and doing laundry. Furthermore, the individual must be able to manage medications as prescribed by a health professional and shop for or otherwise gather the necessities of daily life. The more basic ADL tasks covered by the interRAI assessment tools include performing personal hygiene, being able to move about (whether on foot or in a wheelchair), and toilet use (e.g., using a commode, bedpan or urinal, cleansing oneself, and arranging clothes), among others.

The development of the new IADL Hierarchy and a new IADL-ADL combined scale proceeded through a series of interrelated steps. Existing data were available to permit an examination of individual IADL and ADL item scores among persons receiving home care and those living independently without services. The items could then be subjected to factor analyses to demonstrate the overall continuity across the IADL-ADL continuum. Evidence of the validity of the scales was explored with associative analyses of factors such as a cross-country distributional analysis for persons in home care programs, a count of functional problems across the categories of the hierarchy, an assessment of the hours of informal and formal care received each week by persons in the different categories of the hierarchy, and finally, evaluation of the relationship between cognitive status and the hierarchical IADL-ADL assignments.

The objectives of the analyses here were to: (1) to describe the item distributions in home care and independent living elderly data sets; (2) to demonstrate through a factor analytic procedure how these measures of physical functioning co-existed; (3) to create new IADL and IADL-ADL functional hierarchical summary scales; and (4) to present a series of associative validity findings for the IADL-ADL Functional Hierarchical summary scale (inter-country distributions, problem count, care time, and cognition).

## Methods

### Sample and data

This paper is based on secondary analyses of data from a cross-national sample of community residing persons, almost all of whom are elderly and most of whom receive services from a home-care program, mainly between 2003 and 2008. The dataset, maintained by interRAI, largely consists of computerized home care records provided by governmental service agencies. A cross-country home-care cohort (ADHOC) represents individuals served in several European countries and is supplemented by large home care files from both Finland and Italy. In Canada, home-care data came from the Provinces of Ontario and Manitoba. The home care data in the United States were from Massachusetts, Michigan, Georgia and Louisiana, as well as a sample of independent elders in the community provided by the cross-state COLLAGE voluntary consortium (collageaging.org). COLLAGE collects data from residents of continuing care communities including those who receive home care. The final cohort consisted of persons receiving home care in Hong Kong. In total, interRAI assessments were available for 762,023 persons.

All personal identifiers were removed from the data base, leaving only a code representing the source of the data (e.g., COLLAGE). All assessments were performed by assessors trained in the use of the assessment instrument. The training occurred separately in each country (state or province), but in each instance followed models specified by interRAI [7,8]. Therefore, the reliability of the available data elements can be presumed to be quite good and consistent with those reported previously [9,10,19].

The two assessment instruments used in this study were the interRAI HC (home care) and the interRAI CHA (community health assessment). Both instruments share the same core items. The CHA is designed as a modular instrument to permit about half of the HC questions to be completed for persons receiving only light care. These instruments were designed for use in assessing elderly home care recipients and providing measures relevant to care planning, resource allocation, outcome measurements, and quality assessment. Among about 360 items, are multiple measures of function (ADLs and IADLs) and cognition, as well as the number of hours of formal and informal care received in the 7 days prior to the assessment.

The ADL items used in this analysis include the four performance measures that make up the ADL Hierarchy scale: personal hygiene, locomotion, toilet use, and eating. These items cover a broad spectrum of activities, including items that tend to show decline first (personal hygiene) as well as those where the person retains capacity the longest (eating). Each item is assessed across a 7-category continuum: (0) independent, (1) independent but with some setup help (e.g., laying out clothes), (2) supervision but no direct hands-on support, (3) limited assistance (help but not weight bearing), (4) extensive assistance (weight bearing help but person still performs 50% or more of subtasks), (5) maximal assistance (weight bearing support for more than 50% of subtasks), and (6) total dependence^a^. The set of five IADL capacity items were selected to represent tasks that required the use of different capabilities (both physical and cognitive) and which occur in different locations (in and out of the home). These items include: meal preparation, ordinary housework, managing finances, managing medications, and shopping. Similar to the ADLs, these capacity measures are assessed along a 7-category continuum: (0) independent, (1) setup help only, (2) supervision, (3) limited assistance (help on some occasions), (4) extensive assistance (help throughout task but persons performs less 50% or more of task on own), (5) maximal assistance (help throughout task but persons performs less than 50% of task on own), and (6) total dependence. The choice of “capacity” is quite purposeful in that it calls for the assessor to base his/her assessment on a person’s presumed ability to carry out an activity. Thus the assessment is not biased by social decisions as to who does specific tasks (e.g., the husband or the wife), nor is it biased by societal expectation concerning whether or not an activity is appropriate for a male or a female. In addition, each IADL includes a set of relevant sub-task examples, which can be altered to better reflect the economic reality in a country.

### Analytic approach

The analyses proceeded through a multi-step process. The first analysis presents information on those who receive support in each of the IADL and ADL areas, with a comparison between the typical person receiving home care and a cross-section of elders in the community (as represented in the US COLLAGE cohort). In this and other analyses, due to the enormous size of the samples, we concentrated on differences in the pattern of the distributions and substantiality, as even very small percentage differences between comparison groups of this size would be statistically significant.

Next, for the IADL items, we assess whether there are differences in the structure of inter-item correlations for ethnic subgroups as compared to the broader population values. This assessment is based on KR 20 Alpha internal consistency values. More specifically, does the structure of inter-item relationships for diverse ethnic subgroups fall outside of the normal range? In our view, the IADL tasks are broad in scope and this should not occur, but to address this concern we evaluate the structure of the responses for a number of distinct subsets of elders available in our dataset: blacks in the US, Chinese in Hong Kong, Hispanics in the US, aborigines in Canada, and Japanese in Japan. We first generate KR 20 Alpha values for all home care clients assessed in several US states (Michigan, Mass, Georgia, Louisiana), several Canadian Provinces (Ontario, Manitoba), several European Countries (Germany, Italy, Finland, Iceland), and New Zealand. We next generate KR 20 values for the ethnic subgroups, and then compare the range of observed values.

In the next step, a principal component factor analysis, with a varimax rotation, was completed on the IADL and ADL items in order to understand the interrelationships among the full set of functional measures. Each item was trichotimized so as to have three possible levels: (0) no support from others, (1) some support from others, and (2) totally dependent. This analysis provided an overview of the structure that related these two sets of items. The goal was to draw on these relationships to derive a hierarchical scale that measured performance from independence to total dependence.

Next we evaluated how best to integrate the two scales – the ADL Hierarchy and the new IADL Hierarchy just created – thereby making a single IADL-ADL Functional Hierarchy scale. At the extremes the task is simple. At the independent end of the spectrum of functionality, persons are unlikely to receive support from others in any of the ADLs. Therefore the lower scores on the IADL-ADL Hierarchy could be set based on the IADL Hierarchy. Similarly at the more dependent end of the spectrum, almost all persons could be expected to have each and every one of the IADL deficits. Thus the higher scores on the IADL-ADL Functional Hierarchy scale are based on the higher score of the ADL Hierarchy. At the middle of the spectrum, we looked at the combination of scores from both scales to assign the mid-scale scores. To assist with this analysis, we looked at the count of the number of IADL and ADL problems across the intercepting categories of the IADL Hierarchy and the ADL Hierarchy. As the problem count rose, the IADL-ADL score also rose; when the problem count for adjacent intercepting IADL and ADL cells were about the same they received the identical IADL-ADL Hierarchy score.

In the final set of analyses, we developed descriptive and validation information for the new interRAI IADL-ADL Functional Hierarchy scale. Included is a distributional analysis across the categories of the interRAI IADL-ADL Functional Hierarchy scale by country; and validation crosswalks based on a count of functional problems, an assessment of the hours of informal and formal care received, and finally, an assessment of the cognitive performance distribution across categories (Note, given the large number of available assessments, all comparisons were statistically significant, so the analysis focused primarily on the substantiality of differences).

The data used in this study were provided by the sites which granted authorized data access. All the analyses were approved as secondary analysis by Hebrew SeniorLife Institutional Review Board. All statistical analyses were completed using SPSS version 20.

## Results

The persons in the large cross-country home-care data sample were quite diverse. They averaged 75.8 years of age, with 9.8% of persons 90 years of age or older. As expected, there were more females than males (53.5% vs. 46.5%), 35.4% were married, 44.4% widowed, and 35.2% lived alone. They had a broad array of presenting issues: 38.2% had moderate or more severe difficulty in cognitive decision making ability as measured by the Cognitive Performance Scale - CPS (Morris et al., 1994), while 47.1% had a memory problem.

The most common diseases were hypertension (56.4%), congestive heart failure (16.8%), arthritis (48.5%), and diabetes (28.3%). Conditions of special note included the high prevalence of daily pain (47.7%), shortness of breath (23.2%), a fall in the previous 90 days (32.8%), and an unintended weight loss (11.4%). All in all, as would be expected for a home-care cohort, and especially, one which in some sites explicitly had to be restricted to persons deemed at the nursing home level of care, we saw a variety of challenging problems.

**Figure 1:**
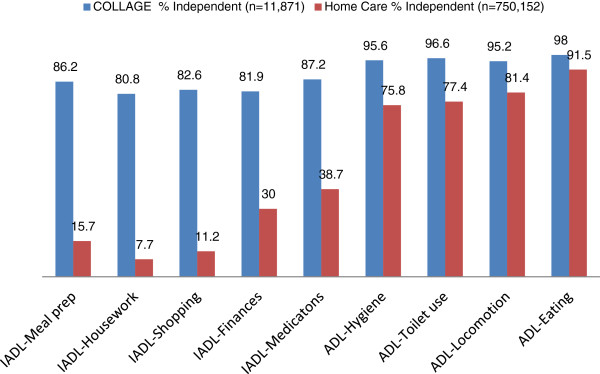
Percent of persons Capable of performing IADLs and ADLs Without Help.

Figure 1 Percent of persons Capable of performing IADLs and ADLs Without Help presents the distributions for the percent of persons in the home care and in the COLLAGE samples who were independent and who did not require the support of others, for each of the IADL and ADL measures. Across the IADL capacity items (the first five in the chart), the vast majority of persons receiving home care did not have the capacity to perform the tasks on their own. Their capacity was best with the two cognitively-based IADLs, i.e., managing finances (30%) and managing medications (38.7%). In the more physically-based IADLs (preparing meals, housework, and shopping), about 90% of persons in home were incapable of performing these tasks on their own.

These rates differed sharply from those seen in the more typical cross-section of community elders as seen in the COLLAGE sample. For these elders, over 80% had the capacity to independently perform each of the five IADL areas. Here, too, even higher percentages of individuals were independent in the four ADLs (personal hygiene, toilet use, locomotion, and eating) – averaging 95% or higher. Similarly for persons in home care, a higher proportion was independent in ADLs, but fewer than we saw in the COLLAGE sample. The rates follow the expected progression from early- to late-loss of ADLs. For the early-loss ADL of personal hygiene, 75.8% are independent; for the mid-loss ADL of locomotion 81.4% are independent; and for the late loss ADL of eating 91.5% are independent.

Prior to assessing the factor structure of the IADL and ADL items, we report on the internal consistency values for the IADL capacity items, as measured by the KR 20 Alpha Reliability statistic. In this step we first generated KR 20 values for all home care clients assessed in large geographic areas (e.g., several US states, several Canadian Provinces, etc.), and here the KR 20 Alpha reliability values ranged from .62 to .86, with a mean of .80 and a median of .83. Next, we generated KR 20 values for the ethnic subgroups (e.g., blacks in the US, Chinese in Hong Kong, Hispanics in the US, aborigines in Canada, and Japanese in Japan), and here the KR 20 values ranged from .71 to .89, with a mean of .82 and a median of .86. There is little difference in these values.

The five IADLs and four ADLs, with each item scored in three categories (independent, requires support, and total dependence), next were subjected to a principal component factor analysis, with a varimax rotation (note, although not shown here, a non-orthogonal solution gave similar results). Separate analyses were completed for the COLLAGE and home care samples. Table 1 presents the first factor for the principal component solution, and in both populations, a unified IADL-ADL continuum was demonstrated. For the varimax solution (not displayed) two factors emerged, an IADL factor and an ADL factor.

**Table 1:** Principal Component Factor Loadings for IADL and ADL items

**Functional variables**	**First principal component factor—COLLAGE**	**First principal component–home care**
IADL-Meal prep	0.838	0.745
IADL-Housework	0.802	0.646
IADL-Shopping	0.797	0.663
IADL-Manage finances	0.739	0.707
IADL-Manage medications	0.789	0.721
ADL-Personal hygiene	0.764	0.722
ADL-Toilet use	0.722	0.743
ADL-Locomotion	0.552	0.704
ADL-Eating	0.650	0.588

The next set of analyses seeks to better understand the hierarchical structure for the IADL measures and those of the ADL measures. Tables 2 and 3 present the relevant information and are structured similarly. The column headings represent the count of the number of areas in which the person is independent, while the rows represent each of the functional items. The cell value is the percentage of persons who remained independent for the function under the condition that there was only the indicated number of areas in which the person was independent. Bolded items in each row indicate the point at which functional independence for that item seemed to have been lost.

**Table 2:** Number of ADLs independent categories with percentage of persons with independence in specific ADL areas

**ADL functional area**	**One independent ADL**	**Two independent ADLs**	**Three independent ADLs**
ADL-Personal hygiene	3.5	24.9	**49.6**
ADL-Toilet use	9.5	**52.7**	88.5
ADL-Locomotion	24.4	**55.6**	78.9
ADL-Eating	**62.9**	67.3	84.0

**Table 3:** Number of IADL independent categories with percentage of persons with independence in specific IADL areas

**IADL functional area**	**One independent IADL**	**Two independent IADLs**	**Three independent IADLs**	**Four independent IADLs**
IADL-Shopping	3.4	6.7	34.9	**77.1**
IADL-Housework	2.0	3.7	13.2	**40.7**
IADL-Meal preparation	4.9	14.8	**70.8**	95.7
IADL-Manage finances	22.1	**83.8**	89.6	94.8
IADL-Manage medications	**67.7**	91.1	91.5	92.1

For ADLs (Table 2), the early loss function is hygiene, the mid-loss functions are toilet use and locomotion, and the late loss function is eating. When there is only one remaining area in which the person is independent, there is a 62.9% chance that it is eating and only a 3.5% chance that it is hygiene. For IADLs (Table 3) a somewhat similar process seems to be at work – all be it a four-step rather than three-step process. The earliest loss IADLs are shopping and housework, followed by meal preparation, managing finances, and managing medications.

By way of background, these underlying hierarchical structures permitted the creation of the two new summary scales – the interRAI IADL Hierarchy and the interRAI IADL-ADL Hierarchy.

As intermediate steps, first a Depend Count variable was created to enumerate the number of areas (from 0 to 5) where the person had no or little capacity to engage in the IADL (they were scored as maximal assistance or total dependence). Second, a Support Count variable identified how many of the five IADL areas required input by others (from set up help to total dependence). Using these two intermediate Counts, the final IADL Hierarchical scale was designed as follows. The scale, like the ADL Hierarchy, has seven levels: (0) Independent (Support Count of 0); (1) Support single area (Depend Count of less than 2 and Support Count of 1); (2) Support in some areas (Depend Count of less than 2 and Support Count of 2 or3); (3) Support in most areas (Depend Count of less than 2 and Support Count of 4 or 5); (4) Dependent in some areas (Depend Count of 2); (5) Dependent in most areas (Depend Count of 3 or 4); (6) Dependent (Depend Count of 5).

**Figure 2:**
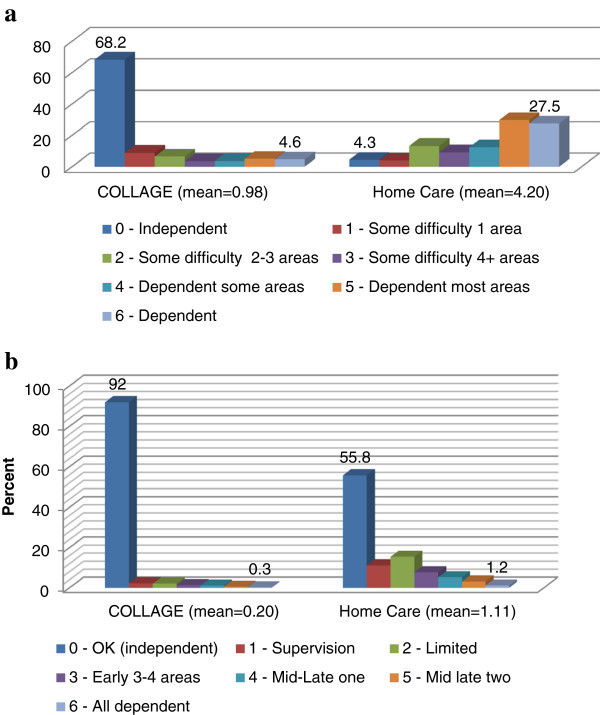
**Percent distributions across hierarchical scales. a.** interRAI IADL Hierarchy Scale Distribution. **b.** interRAI ADL Hierarchy Scale Distribution.

Figure 2a interRAI Hierarchy Scale Distribution and b interRAI ADL Hierarchy Scale Distribution display the distributional properties of the interRAI IADL Hierarchy and interRAI ADL Hierarchy. For the interRAI IADL Hierarchy (Table 2a), the broader sample of elders in the community (as represented by COLLAGE) classifies the majority of persons into the independent category (68.2%). At the same time, there are persons in all seven categories of the scale, but only 9.6% fall into the two most dependent scale categories. For persons receiving home care, the distributions are quite different; 4.3% fall into the independent category and 3.9% are in the single support category. At the other extreme, 57.2% are in the two most dependent categories. Persons receiving home care tend to have multiple problems, and over half have three or more IADL areas in which they are fully dependent on others. For the interRAI ADL Hierarchy (Table 2b), the pattern is somewhat similar. For the broader sample of elders in the community (as represented by COLLAGE), the vast majority of persons fall into the independent category (92%). At the same time, there are persons in all seven categories of the scale, with a small number (0.3%) in the most dependent scale category. For persons receiving home care, the interRAI ADL Hierarchy distributions are different, 55.8% fall into the independent category, 11.2% are in the supervision category, and 15.5% are in the limited support category. At the other extreme, 1.2% are in the most dependent category.

The next scale – the interRAI IADL-ADL Functional Hierarchy Scale – is created through an amalgamation of the ADL Hierarchy and IADL Hierarchy scales. Of note, the inter-scale correlation is .50 in the home-care sample and .57 in the COLLAGE sample, suggesting a reasonable crosswalk between the two scales. The IADLs and ADLs have been shown to follow a relatively consistent hierarchical pattern, the items factor together, and the scale construction process followed the model used in creating the IADL Hierarchy. The high scores on this scale are based on high scores on the ADL Hierarchy, while the low scores are based on low scores on the IADL Hierarchy; and the middle categories are based on a cross walk of the ADL Hierarchy and IADL Hierarchy. Table 4 presents the scoring rules for this new functional scale, where a score of “0” is Independent and a score of “11” is Dependent. Our assignment within the mid-range of the new scale was based on scores on the count of the total areas of IADL and ADL support – shown below.

**Table 4:** Scoring rules for the interRAI IADL-ADL performance hierarchy – based on IADL hierarchy (row) and ADL Hierarchy (column) – 0 = Independent --- 11 = Dependent

	**ADL – 0**	**ADL – 1**	**ADL – 2**	**ADL – 3**	**ADL – 4**	**ADL– 5**	**ADL – 6**
IADL – 0	0	0	2	8	9	10	11
IADL – 1	1	1	2	8	9	10	11
IADL – 2	2	2	6	8	9	10	11
IADL – 3	3	5	6	8	9	10	11
IADL – 4	3	5	6	8	9	10	11
IADL – 5	4	5	6	8	9	10	11
IADL – 6	5	7	7	8	9	10	11

**Figure 3:**
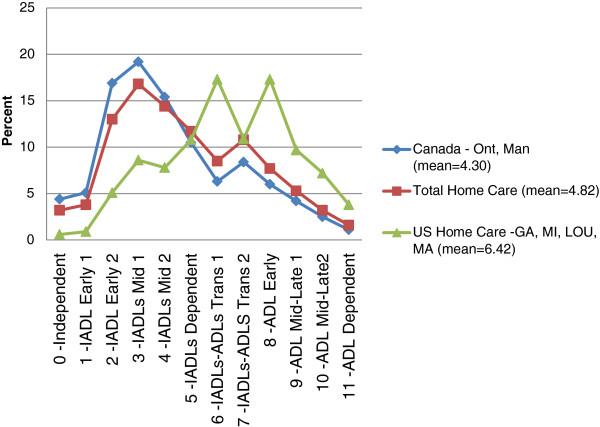
interRAI IADL-ADL Functional Hierarchy Distribution.

Figure 3 interRAI IADL-ADL Functional Hierarchy Distribution displays the scores for the home care sample on the interRAI IADL-ADL Functional Hierarchy scale. Five distributions were evaluated – overall and four country specific groupings (four USA states, two Canadian provinces, three Europe country groups, and Hong Kong). However, for the sake of space, only three are displayed here: the overall average for all persons receiving home care in the interRAI data holdings, and the two outlier countries with the lowest and highest mean score on the interRAI IADL-ADL Performance Hierarchy (Canada and the US). All categories, from independent to dependent, are represented in each of the home care samples, although the distributions are significantly different. Of note, home care clients from the USA were less likely to fall into the five most independent categories (23% for US home-care clients vs. 63% for Canadian home-care clients) and more likely to have more severe ADL problems as seen in scores of 8–11 (38% for US home-care clients vs. 13.8% for Canadian home-care clients and 17.8% for the total home-care cohort).

The final set of validation analyses relate the interRAI IADL-ADL Functional Hierarchy to a series of external measures: a count of thirteen IADL and ADL areas where the persons received support from others (the criterion measure to help specify the middle-categories of the IADL-ADL Hierarchy); informal care hours in the past week; formal care hours in the past week; total care hours in the past week; cognitive performance as assessed with the CPS [20].

**Figure 4:**
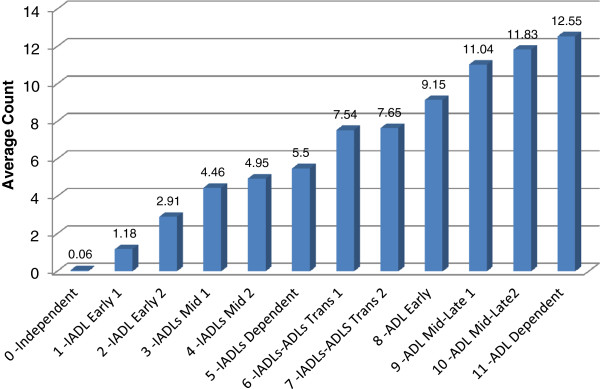
Average Count of total Areas of IADL and ADL Support (max = 13, mean = 5.86; eta = .834).

Figure 4 Average Count of total Areas of IADL and ADL Support (max = 13, mean = 5.86; eta = .834) examines how the count of IADL and ADL areas where support is received increases across the categories of the IADL-ADL Functional hierarchy. The mean count of areas in which support is provided rises steadily from 0.06 areas for those who are independent to a mean of 5.5 for persons in the middle of the scale range, to 12.6 for persons at the high end of the scale -- who are dependent in ADLs. This progression is also reflected in the high correlation (an eta of .834).

**Figure 5:**
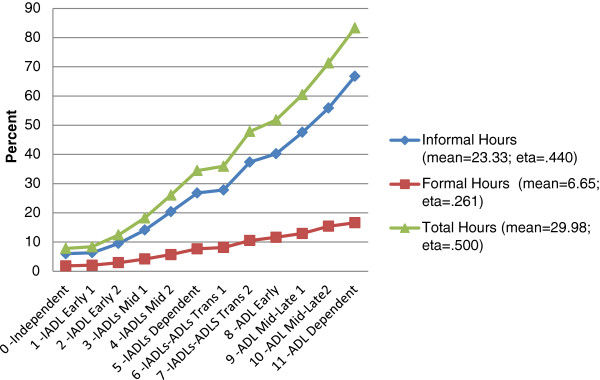
Average Hours of Weekly Support Across the Categories of the interRAI IADL-ADL Functional Hierarchy Scale.

Figure 5 Average Hours of Weekly Support Across the Categories of the interRAI IADL-ADL Functional Hierarchy Scale examines how a value on the IADL-ADL Functional Hierarchy scale translates into the hours of care received in the prior week. For informal care, there is a steady progression across all of the categories, mimicking the progression seen in the previous figure. For persons who are in the independent category (0) friends and relatives provide an average of 6.0 hours of support a week (whether they need it or not); persons in the middle of the scale range (with a score of 6) receive an average of 27.8 hours of informal care a week (approximately 4 hours a day); persons in the most dependent category receive an average of 66.7 hours of informal care per week (i.e., 9.5 hours a day).

The formal care profile is quite different from that of informal care. The weekly hours of care begin at a very low level – an average of 1.8 hours a week for persons in the independent category of the IADL-ADL Functional Hierarchy. There is a slight increase as persons lose capacity – with a jump to 8.1 hours a week for persons in category six of the functional hierarchy. As would be expected, persons in the most dependent category of the functional hierarchy receive the most formal care – an average of 16.6 hours per week. When the formal care and informal care are brought together, the overall profile of increasing care hours follows the informal profile – 80% of all care is provided informally and as functional capacity decreases both formal and informal care hours increase as well. As persons lose the ability to perform these activities on their own, informal systems increase their commitment dramatically. Home-care clients also benefit from formal support resources, but they appear to be primarily supplements to the informal support infrastructure available to the person. Although not shown in the chart, where formal care is provided, there is a 92% likelihood that informal care is also being provided.

**Figure 6:**
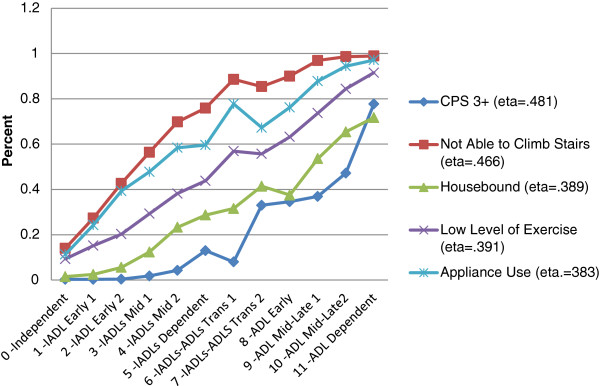
Cognitive and Functional Challenges Across the Categories of the interRAI IADL-ADL Functional Hierarchy.

Figure 6 Cognitive and Functional Challenges Across the Categories of the interRAI IADL-ADL Functional Hierarchy displays the proportion of persons who have an underlying problem in five different cognitive and performance areas that shown to be related to functional status. The cognitive measure is a score of three to six for the Cognitive Performance Scale, representing significant cognitive impairment. Persons with a functional hierarchy score of less than seven are largely in the more independent CPS categories. About a third of the persons in hierarchy categories from 8–10 have more severe cognitive problem. This rate rises to 47% for persons in category 10, and 78% for persons in category 11.

The functional measures in Figure 6 include an inability to climb stairs, the use of an assistive device (e.g., cane), being housebound, and a low level of exercise. All display the same pattern – an increase restricts performance across the categories of the interRAI IADL-ADL Functional Hierarchy.

## Discussion

Using items from interRAI’s suite of assessment instruments, two new functional scales have been added to the interRAI set of measures – the interRAI IADL Hierarchy Scale and the interRAI IADL-ADL Functional Hierarchy Scale. They join the widely used interRAI ADL Hierarchy Scale to cover the spectrum of IADL and ADL challenges faced by persons in the community. The interRAI functional assessment items used to create these scales have high reliability, have been used in a wide variety of applications, and have proven validity.

The development of the scales exploits the sequential loss of IADL and ADL functions. For ADLs, it replicates earlier work that suggests that there is an early, mid, and late loss ADL decline paradigm. The early loss ADLs represent the first personal activities of daily living in which one is likely to receive help from others, which in our item set is represented by personal hygiene. At the other extreme, for the late-loss ADLs where one is most likely to retain some personal involvement in the activity, eating was that activity. For the IADL capacity items, a somewhat similar hierarchical loss pattern was observed: the early loss IADLs are shopping and housework, followed by meal preparation, managing finances, and managing medications.

The distribution of home-care clients across the categories of the IADL-ADL Functional Hierarchy Scale differs by country. While few home care clients were in the two most independent categories of the 12-point scale, clients in US sites were more likely than clients in Canada or Europe to have multiple IADL deficits or early loss ADL problems.

In a series of comparative analyses, the interRAI IADL-ADL Functional Hierarchy Scale was sensitive to care and other issues. The mean areas of support by others went up in a linear function across the categories of the scale, rising from 0.6 areas for those who were independent (scale point 0) to 12.55 areas for those who were totally dependent in ADLs (scale point 11). There is a similar steady progressive increase in the hours of informal and total care received across the categories of the scale – from 6.2 hours of informal care a week at the low end of the scale, to an average of 65.8 hours of informal care a week at the high end of the scale. Of the other measures contrasted against the scale, similar steady progressions in problem levels were observed for not being able to climb stairs, engaging in a low level of exercise, using appliances, and being housebound. In the cognitive domain the relationship differed slightly. Persons in the first eight score categories of the scale are largely in the three most independent categories of the CPS. This proportion drops progressively from scale score 8 through 12.

## Conclusion

In review of these results, the value of an appropriate integrated IADL and ADL functional assessment tool cannot be overstated. The loss in these areas follows a general hierarchical pattern and with the interRAI IADL-ADL Functional Hierarchy Scale this progression can be reliably and validly assessed. The assessment burden involves only nine items: four ADLs (hygiene, toilet use, locomotion, and eating) and five IADLs (meal preparation, housework, shopping, managing finances, and managing medications). This scale can be scored independently using this limited item set or it can be constructed automatically from a number of the interRAI assessments (e.g., the interRAI Home Care or interRAI Community Health Assessment). With the global use of the interRAI tools, wide use of these new scales can be expected. The validation findings in this paper demonstrate a dramatic relationship between increasing functional loss and the amount of care received, the cognitive status, of the person and leaving home.

As one example of the need to bring functioning into health areas where it is often neglected, the success or failure of a particular intervention in the field of oncology often is determined by comparing the duration of life in the control and the treatment groups with no or little consideration given to the functional profile of the patients. On occasion the cost of the intervention may be provided so that the cost per additional month of life can then be calculated. But older persons especially may need to have more than the duration of life measured. As stated by Balducci [21], the need to assess quality of life (QOL) in older patients with cancer is essential and, as this author noted, there might be a “trade-off between QOL and survival.”

However, formal standardized functional assessments are rarely used to judge the merits of an intervention following a specific therapy. A recent review of the summaries of some 356 articles directed to “functional outcomes” in oncology was undertaken on the PubMed. One study directed to the management of cachexia in cancer patients endorsed the idea of measuring overall physical activity to determine the merits of an intervention [22]. A few articles did highlight general functional issues although these were usually concerned with initiatives to improve palliative care. Very few articles were directed to organ-specific function, such as improvement in visual acuity or the beneficial changes following a specific treatment of tumors which had damaged the spinal column. None provided detailed assessments of ADLs and IADLs or how these differed in those treated in one way and another, and we see the new IADL-ADL Hierarchy scale as a tool that could be easily incorporated into this type of health initiative.

In order to achieve a better understanding of the merits of a particular intervention, especially in the elderly population -- and not just in the field of oncology, detailed functional assessments should be mandatory when such interventions are proposed and studied. This is highlighted by the fact that older persons are likely to have a host of other diseases which may make a specific intervention that can extend life only a small amount not only costly but undesirable.

This issue is likely to become an ever more important one as the specialties of medicine become increasingly isolated from each other. Many elders require the services of multiple specialists. However without a primary care physician who can oversee the care of each unique patient and assess overall functional capability, the outcome of care is not likely to be optimal.

## Endnote

^a^There is one additional ADL code option, an 8 which signifies that the activity did not occur during the entire period. When observed, this response is recoded to a 6, total dependence.

## Competing interests

JNM, KB, BEF, and SK are members of the not-for-profit interRAI international group. JNM, BEF, and EH participate in the US bases COLLAGE non-for-profit organization.

## Authors’ contributions

JNM led the study design, analysis and interpretation of data, and preparation of the manuscript. KB, BEF, KS, and EH collaborated in interpretation of data and revision of the manuscript. The final version of the manuscript was revised and approved by all authors.

## Pre-publication history

The pre-publication history for this paper can be accessed here:

http://www.biomedcentral.com/1471-2318/13/128/prepub
